# Polysaccharides isolated from *Bangia fuscopurpurea* induce apoptosis and autophagy in human ovarian cancer A2780 cells

**DOI:** 10.1002/fsn3.2621

**Published:** 2021-10-10

**Authors:** Jingna Wu, Changhong Lin, Xiaoting Chen, Nan Pan, Zhiyu Liu

**Affiliations:** ^1^ Xiamen Key Laboratory of Marine Medicinal Natural Products Resources Xiamen Medical College Xiamen China; ^2^ Fujian Universities and Colleges Engineering Research Center of Marine Biopharmaceutical Resources Xiamen Medical College Xiamen China; ^3^ The First Affiliated Hospital of Xiamen University Xiamen China; ^4^ Fisheries Research Institute of Fujian Xiamen China

**Keywords:** apoptosis, autophagy, *Bangia fuscopurpurea*, ovarian cancer, polysaccharide

## Abstract

Although ovarian cancer is common, its prognosis remains poor because of drug resistance and early metastasis. Polysaccharides extracted from *Bangia fuscopurpurea* (BFP) are potential anti‐cancer agents, but the mechanisms underlying their effects in human ovarian cancer remain unclear. Here, we investigated the mechanisms of action of BFP polysaccharides in A2780 ovarian cancer cells using cell migration, invasion, apoptosis, and autophagy assays. Transwell assays indicated that BFP inhibited cell migration and invasion. Flow cytometry analysis showed that BFP treatment induced apoptosis and reactive oxygen species production, while significantly reducing mitochondrial membrane potential. Reverse transcription–polymerase chain reaction and Western blot analyses revealed changes in the expression of apoptosis‐ and autophagy‐related cellular mRNAs and proteins, respectively, following BFP treatment for 24 h. Transmission electron microscopy revealed that BFP induced autophagy in A2780 cells. These findings demonstrate that BFP may be useful for developing functional foods for cancer therapy.

## Graphical abstract

BFP suppresses human ovarian cancer A2780 cell migration, and invasion. BFP induces mitochondria‐mediated apoptosis and autophagy in A2780 cells. BFP may possess anti‐ovarian cancer properties in vitro.

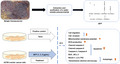

## 1 INTRODUCTION

Annually, 240,000 women are diagnosed with ovarian cancer worldwide with a 5‐year survival rate of <45% due to the insidious early symptoms and late detection, thereby highlighting it as the 7th and 8th most commonly reported cancer and cause of cancer‐related death among women, respectively (Webb & Jordan, 2017). Cytoreductive surgery is the standard treatment for primary ovarian cancer; in advanced stages, adjuvant platinum/taxol chemotherapy and treatment with anti‐angiogenic agents such as bevacizumab are used (Sun et al., 2016). However, chemotherapeutic drug resistance and early metastasis render poor prognosis in ovarian cancer (Li, Hong, et al., 2018). Thus, more effective therapeutic agents are urgently needed.

Many natural products, including various phytochemicals, have proven to exert anti‐cancer activities against ovarian cancer without negatively affecting normal cells. Polysaccharides, for example, can suppress the migration and invasion of ovarian cancer cells. Specifically, selenium‐enriched polysaccharides extracted from *Pyracantha fortuneana* inhibit their migration by suppressing the β‐catenin signaling pathway (Sun et al., 2016). Furthermore, crude polysaccharides extracted from *Rosa roxburghii* Tratt suppress their migration and invasion (Chen et al., 2014). Ovarian cancer cell migration and invasion constitute a complex and multi‐staged process that depends on the activity of several mediators. The most effective strategy for cancer treatment is the induction of tumor cell apoptosis and maintenance of a balance in the number of cells. Notably, autophagy is a key regulatory factor in cell death (Zhu et al., 2018), while apoptosis is closely related to chromatin condensation, nuclear disruption, DNA fragmentation, and caspase activation. However, autophagy is known to be compromised by nutrient deficiency or stress (Peng et al., 2018).

Considering the attractive prospects of natural products in the treatment of ovarian cancer, we previously purified a homogeneous polysaccharide derived from *Bangia fuscopurpurea* (BFP), a primitive red alga commonly found in the rocky intertidal zone, and evaluated its antitumor effect in vitro (Wu et al., 2021). BFP substantially inhibited the growth of ovarian cancer cells, providing insights into the mechanism by which BFP participated in tumor progression; however, the underlying mechanisms by which BFP treatment sensitizes cancer cells to death remain unclear. Therefore, in this study, we investigated these mechanisms and the role of BFP in suppressing migration, cell cycle arrest, invasion, and in inducing apoptosis and autophagy in A2780 cells. Our results support the potential applicability of BFP as an alternative agent for cancer therapy.

## 2 MATERIALS AND METHODS

### 2.1 Reagents and antibodies

*Bangia fuscopurpurea* was purified from *B. fuscopurpurea* as per methods previously reported. Briefly, dried *B. fuscopurpurea* were extracted twice with distilled water at 100°C for 4 h. The combined aqueous extract was precipitated by the addition of ethanol to a final concentration of 85% (*V*/*V*) at 4°C overnight. After centrifugation, the separated precipitate was deproteinated by an enzymatic hydrolysis method (neutral protease, 56°C, 4 h). Subsequently, the crude polysaccharides were fractionated by a DEAE‐Sepharose Fast Flow (50 mm × 500 mm) column, and two fractions were collected. The fraction with potent antitumor activities was further purified using a Sephacryl S‐100 High‐Resolution (3.0 cm × 120 cm) column to yield the target polysaccharide BFP. Finally, characterization of BFP was carried out using GC‐MS, partial acid hydrolysis, methylation, and one‐/two‐dimensional NMR. (Wu et al., 2021). The human ovarian cancer cell line A2780 was purchased from Meixuan Biological Science Co. Ltd.; the Dulbecco's modified Eagle medium (DMEM) was purchased from Corning Inc.; fetal bovine serum (FBS) was purchased from Gibco; N‐acetylcysteine (NAC) was purchased from Sigma‐Aldrich; antibodies against glyceraldehyde‐3‐phosphate dehydrogenase (GAPDH; ab8245, 40 kDa), cleaved caspase‐3 (ab2302, 17 kDa), cleaved caspase‐9 (ab2324, 34 kDa), caspase‐3 (ab13847, 34 kDa), caspase‐9 (ab32539, 46 kDa), BAX (ab32503, 21 kDa), LC3 (ab48394, 15 kDa), Beclin‐1 (ab210498, 52 kDa), BCL2 (ab32124, 26 kDa), and P62 (ab109012, 62 kDa) were purchased from Abcam; and the secondary antibody (HRP‐conjugated goat anti‐mouse IgG, D110087) was purchased from BBI. All other chemicals and reagents were of analytical grade.

### 2.2 Transwell assay

A2780 cells (5 × 10^4^ cells/well) were subjected to treatment with 0.01 µg/mL taxol (positive control, PC), BFP (1, 3, and 9 µg/mL), or DMEM (negative control, NC) for 24 h and were analyzed using a Transwell assay to evaluate cell migration and invasion. Cells were resuspended in a serum‐free medium and seeded into the upper chamber of the Transwell insert (Corning Inc.) with an 8‐µm pore‐size filter membrane at a density of 5 × 10^5^ cells/mL (100 µL/chamber), whereas 500 µL of a conditioned medium containing 20% FBS was added to the lower chamber. The cell invasion assay was performed in a manner similar to the cell migration assay, except that the upper chamber was coated with Matrigel (Corning Inc.) in the former method. The cells were incubated at 37°C in an atmosphere of 5% CO_2_ for 12 h. Cells in the upper chamber were removed using cotton swabs, whereas migrated/invaded cells on the bottom side of the membrane were subjected to fixation in 4% paraformaldehyde for 10 min, and were stained with crystal violet solution for 5 min, following which they were counted in five randomly selected microscopic fields (200×) per well.

### 2.3 Cell cycle analysis

A2780 cells seeded in a 6‐well plate (2.5 × 10^5^/mL) were subjected to treatment with 2 mL BFP (1, 3, and 9 µg/mL) and taxol (0.01 µg/mL, PC group) and incubated for 24 h. The cells were collected and subjected to fixation for 12 h with pre‐cooled 70% ethanol at 4°C and were further harvested by centrifugation at 1,000 g for 5 min, after which the supernatant was discarded. The cells were resuspended in 1 mL pre‐cooled phosphate‐buffered saline (PBS) and collected by centrifugation. Next, 50 mL propidium iodide (PI) staining solution was added according to the cell cycle and apoptosis kit (Beyotime) instructions, and the mixture was incubated in the dark at 37°C for 10 min before subjection to analysis using the FACS Calibur flow cytometer (BD Biosciences).

### 2.4 Apoptosis analysis

A2780 cells were harvested after treatment with BFP, NAC, or taxol for 24 h, and then stained with Annexin V‐fluorescein isothiocyanate (FITC; 5 μL) and PI (10 μL) for 10 min at 20–25°C in the dark. Apoptosis was determined using the Annexin V‐FITC apoptosis detection kit (Beyotime) instructions. The proportion of apoptotic cells was determined by flow cytometry.

### 2.5 Mitochondrial membrane potential (MMP) determination

A2780 cells were subjected to treatment with BFP or taxol for 24 h, harvested and subjected to washing steps twice, resuspended in 1 µmol/L DiO staining solution (Solarbio), and then incubated at 37°C for 10 min, according to the cellular membrane potential assay kit (Solarbio) instructions. The MMP was determined by flow cytometry.

### 2.6 Measurement of reactive oxygen species (ROS) generation

As per the reactive oxygen detection kit (Solarbio) instructions, A2780 cells were harvested after treatment with BFP or taxol for 24 h. The culture medium was removed, and the cells were subjected to washing steps twice with PBS. Next, the cells were incubated with 10 μmol/L DCFH‐DA (2′,3′‐dichlorodihydrofluorescein diacetate) probe at 37°C for 20 min. At the indicated time points, the culture medium was discarded, and the cells were subjected to washing steps three times with PBS. ROS production was measured by flow cytometry.

### 2.7 Transmission electron microscopy (TEM)

A2780 cells were collected after subjection to 24 h of treatment with BFP or taxol and fixation in 2.5% glutaraldehyde (1 mL) at 4°C overnight; thereafter, the cells were subjected to washing steps 4 times with PBS, and then fixed with 1% osmic acid at 4°C for 2 h. The cells were then dehydrated using a graded ethanol series from 50% to absolute ethanol, infiltrated with acetone, and embedded in Araldite 812. Sections (70 nm) were cut, stained with uranyl acetate and lead citrate, and observed by TEM (FEI).

### 2.8 Quantitative reverse transcription–PCR (qRT–PCR)

Cells were harvested after incubation with different doses of BFP and taxol for 24 h. Total RNA extraction was performed following the MagBeads Total RNA Extraction Kit instructions (Meixuan Biological Science Co. Ltd.). Primers (Table 1) were designed using Primer Premier 5.0. The reactions were conducted using the ViiA7 RT‐PCR thermocycler (Applied Biosystems) and SYBR Premix Ex Taq (Meixuan Biological Science Co. Ltd.) according to the manufacturer's instructions. The thermocycling conditions were reverse transcription at 50°C for 10 min and pre‐denaturation at 95°C for 10 min, followed by 45 cycles of 95°C for 15 s and 60°C for 30 s. Quantification data were evaluated using the Light Cycler analysis software. The comparative 2^−ΔΔCt^ method was performed using *GAPDH* mRNA as an endogenous reference. The results were presented as the ratios of target mRNA to *GAPDH* mRNA.

**TABLE 1:** Quantitative polymerase chain reaction primer sequences

Gene	Forward primer (5′–3′)	Reverse primer (5′–3′)
*CASP3*	TGGAACCAAAGATCATACATGGAA	TTCCCTGAGGTTTGCTGCAT
*CASP9*	GCCCTCCCCACTAAGACCTA	TGTCCTTACACTGGAAGAAGAGA
*BCL2*	CATGTGTGTGGAGAGCGTCA	CACTTGTGGCTCAGATAGGCA
*BAX*	TGATGGACGGGTCCGGG	GGAAAAAGACCTCTCGGGGG
*LC3*	TTCCGAGTTGCTGACTGACC	CCCTTGTAGCGCTCGATGAT
*P62*	TGTGAGCTAGCACTGTGGAAA	ATCATCCGTCTCTTCCCCGA
*BECN1*	GGGCTCCCGAGGGATGG	GCTGTTGGCACTTTCTGTGG
*GAPDH*	GCACCGTCAAGGCTGAGAAC	TGGTGAAGACGCCAGTGGA

### 2.9 Western blot analysis

Western blotting was used to detect the expression of apoptosis‐ and autophagy‐related proteins after the cells were subjected to treatment with BFP or taxol. Cell proteins were extracted and measured using a BCA protein assay kit (Beyotime), after which proteins were separated (20 µg) on a 12% SDS‐PAGE gel and transferred onto a polyvinylidene fluoride membrane (Bio‐Rad Instruments, Hercules, CA, USA). The membrane was blocked using 5% bovine serum albumin for 2 h, washed with PBS, and incubated at 4°C overnight with primary antibodies against GAPDH, caspase‐3, cleaved caspase‐3, caspase‐9, cleaved caspase‐9, BAX, LC3, Beclin‐1, BCL2, or P62 (1:1,000). The membrane was washed again with PBS and incubated with a horseradish peroxidase‐conjugated secondary antibody (1:5,000; Abcam) at 25°C for 2 h. Finally, the membrane was washed again with PBS, subjected to reaction with enhanced chemiluminescence reagent (ELC, Millipore), and exposed to an X‐ray film to detect the signals.

### 2.10 Statistical analysis

All experiments were conducted in triplicate, and the data were calculated as mean ± standard deviation (SD). Student's *t* tests were conducted using the SPSS 22.0 software (SPSS, Inc.) to detect significant differences between treatments, with *p* < .05 considered statistically significant.

## 3 RESULTS

### 3.1 Effect of BFP treatment on A2780 cell invasion and migration

**FIGURE 1:**
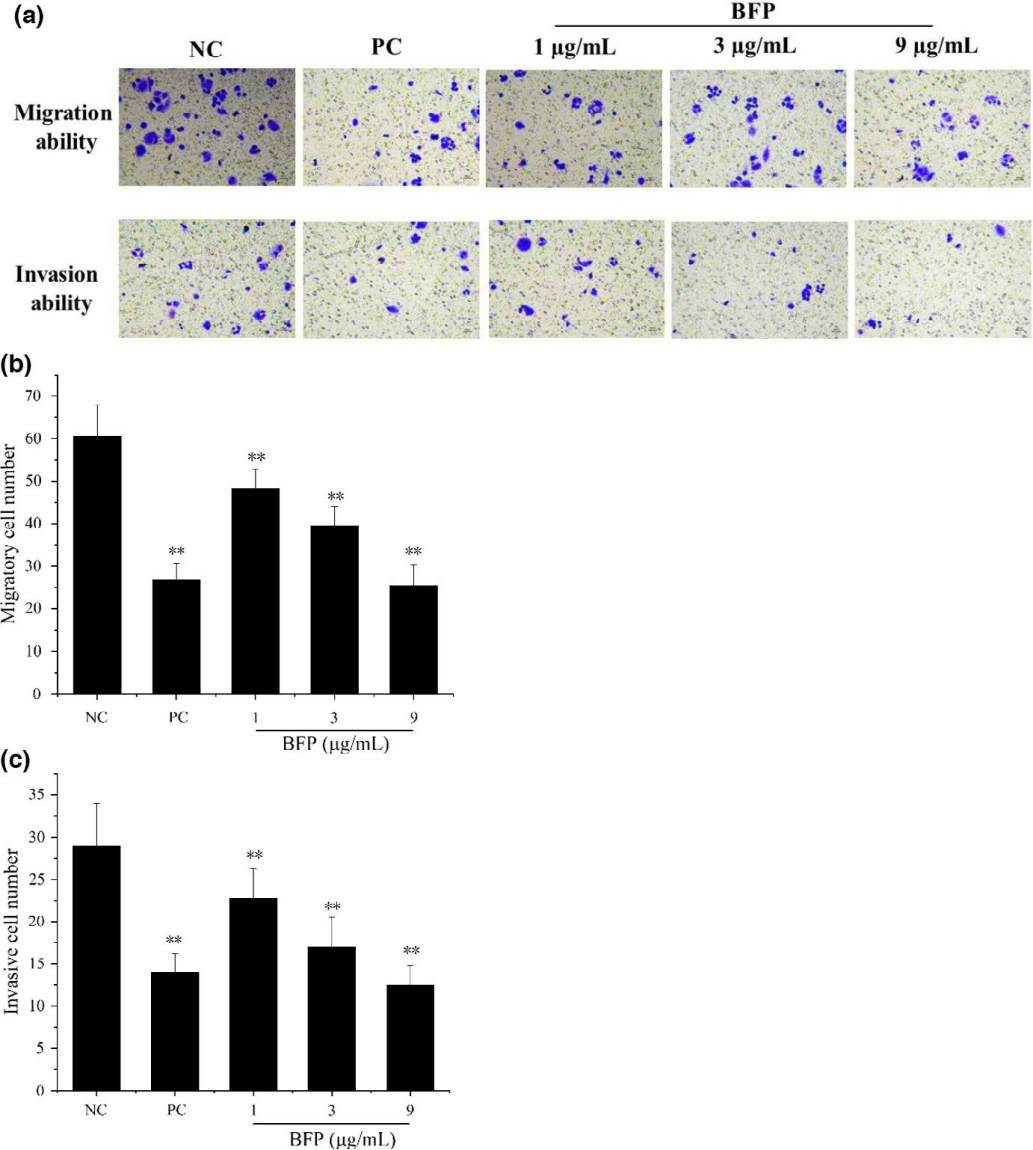
*Bangia fuscopurpurea* (BFP) treatment abrogated migration and invasion capacity of A2780 cells (200×). (a) A2780 cells were incubated with 0.01 µg/mL taxol (positive control, PC), with different concentrations of BFP (1, 3, and 9 µg/mL), or with the Dulbecco's modified Eagle medium (DMEM; negative control, NC) for 24 h. Next, cell migration and invasion were detected in a Transwell assay. Quantification of migrated (b) and invaded (c) cells. *p < .05 and **p < .01 versus. NC group (mean ± SD, *n* = 3)

After treatment with BFP (1, 3, and 9 µg/mL), A2780 cell migration and invasion were significantly reduced in a dose‐dependent manner compared with the NC group (*p* < .01) (Figure 1).

### 3.2 Effect of BFP treatment on cell cycle in A2780 cells

**FIGURE 2:**
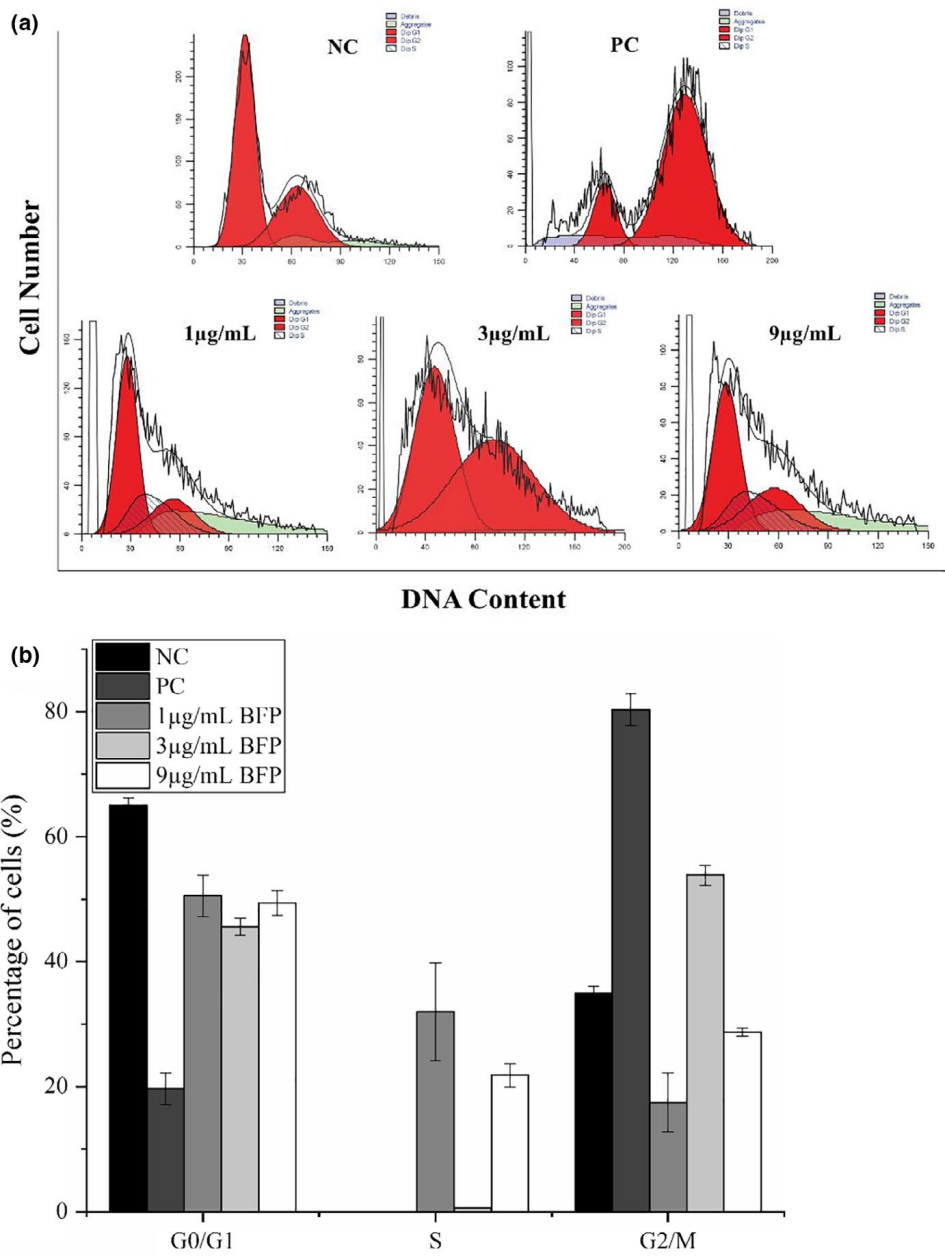
Effects of BFP treatment on cell cycle progression in A2780 cells determined using flow cytometry. A2780 cells were subjected to treatment with 0.01 µg/mL taxol (positive control, PC) and BFP (1, 3, and 9 µg/mL), and a negative control (NC) group was established. (a) Representative graph of (propidium iodide) PI‐stained cells sorted using flow cytometry. (b) Quantification of the percentage of cells in various phases of the cell cycle using flow cytometry

One of the key requirements for cell growth and proliferation is a normal cell cycle progression. Growth inhibition is often accompanied by cell cycle arrest (Ma et al., 2020). To further investigate the growth inhibitory effects of BFP in A2780 cells, cells in each phase of the cell cycle were calculated and compared with the total cell number. Compared with cells in the NC group, cells subjected to treatment with taxol (PC) underwent arrest in the G2/M phase. BFP exhibited different effects on the different cell cycle phases; however, no considerable dose relationship could be observed (Figure 2).

### 3.3 Effect of BFP treatment on apoptosis in A2780 cells

**FIGURE 3:**
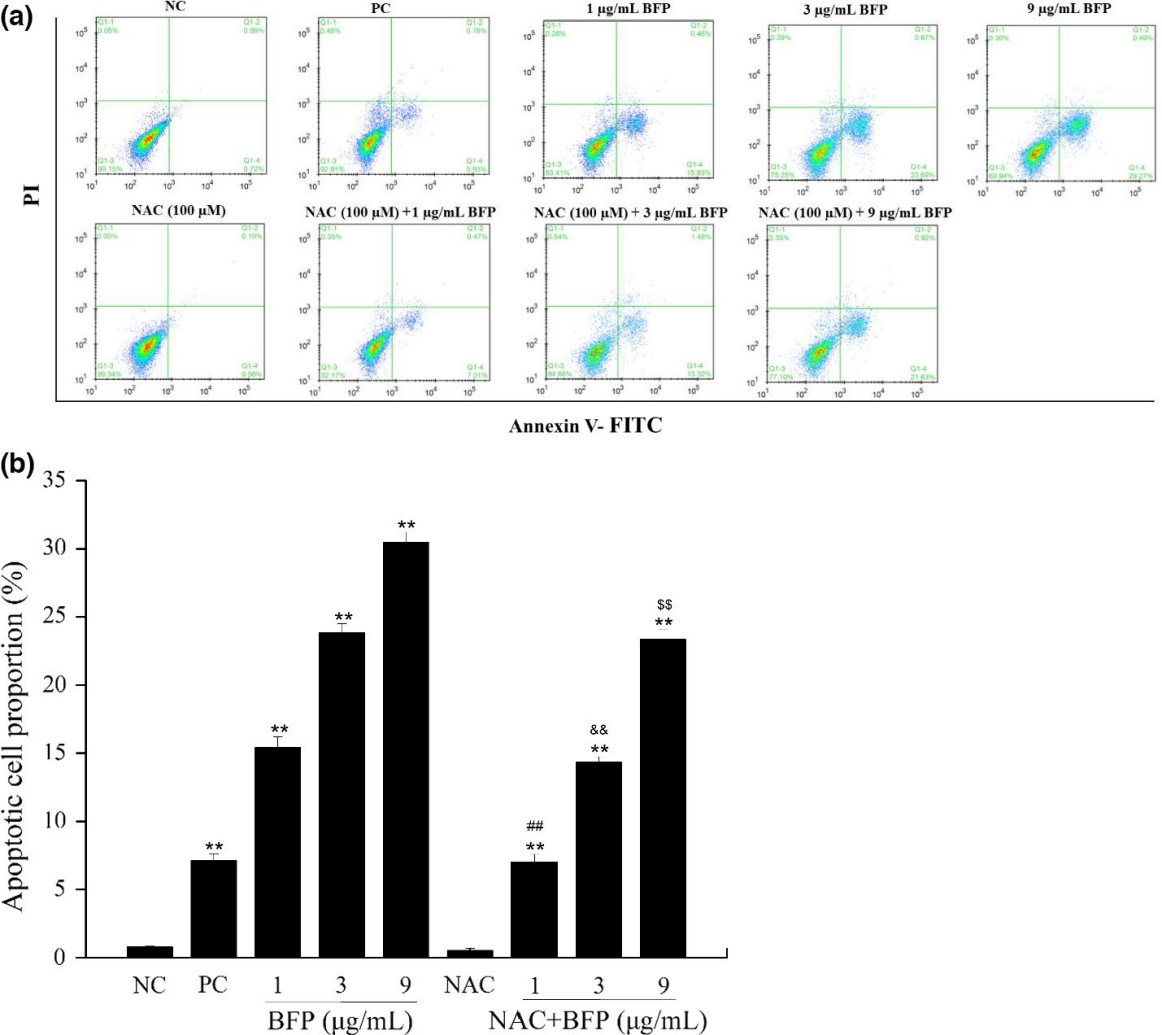
BFP treatment induced apoptosis. A2780 cells were subjected to treatment with 0.01 µg/mL taxol (positive control, PC), BFP (1, 3, and 9 µg/mL), NAC (100 µM), NAC (100 µM) + BFP (1, 3, and 9 µg/mL), and a negative control (NC) group was established. (a) Cell apoptosis was evaluated using flow cytometry after staining with Annexin V‐FITC/PI (V‐fluorescein isothiocyanate). Cells in quadrants Q1‐1, Q1‐2, Q1‐3, and Q1‐4 represent necrotic, late apoptotic, viable (live), and early apoptotic populations, respectively. (b) Quantification of apoptotic cells. **p < .01 versus. NC group (mean ± *SD*, *n* = 3), ^##^ *p* < .01 versus. BFP (1 µg/mL) group (mean ± SD, *n* = 3), ^&&^ *p* < .01 versus. BFP (3 µg/mL) group (mean ± *SD*, *n* = 3), ^$$^ *p* < .01 versus. BFP (9 µg/mL) group (mean ± SD, *n* = 3)

To determine whether the reduced cell proliferation following BFP treatments occurred due to apoptosis, Annexin V‐FITC/PI staining was performed to quantify the apoptotic populations and cell death. BFP treatment significantly increased A2780 cell apoptosis at the tested concentrations, from 0.77% ± 0.09% in the NC group to 15.40% ± 0.79% (*p* < .01), 23.84% ± 0.67% (*p* < .01), and 30.48% ± 0.74% (*p* < .01) in the 1, 3, and 9 µg/mL BFP treatment groups, respectively (Figure 3). Furthermore, to identify whether apoptosis induced by BFP treatment was ROS‐dependent, the A2780 cells were treated with BFP (1, 3, and 9 µg/mL) in the presence of an ROS inhibitor (NAC, 1 μM). As a result, BFP‐induced apoptotic was suppressed in A2780 cells when treated with 1 μM ROS inhibitor (NAC), and the cell apoptosis was reduced by 8.39% (*p* < .01), 9.52% (*p* < .01), and 7.13% (*p* < .01) in the 1, 3, and 9 µg/mL BFP treatment groups, respectively (Figure 3). The presented findings provide novel evidence that BFP induces apoptosis in A2780 cells, which involved the pathway of ROS.

### 3.4 Effect of BFP treatment on MMP in A2780 cells

**FIGURE 4:**
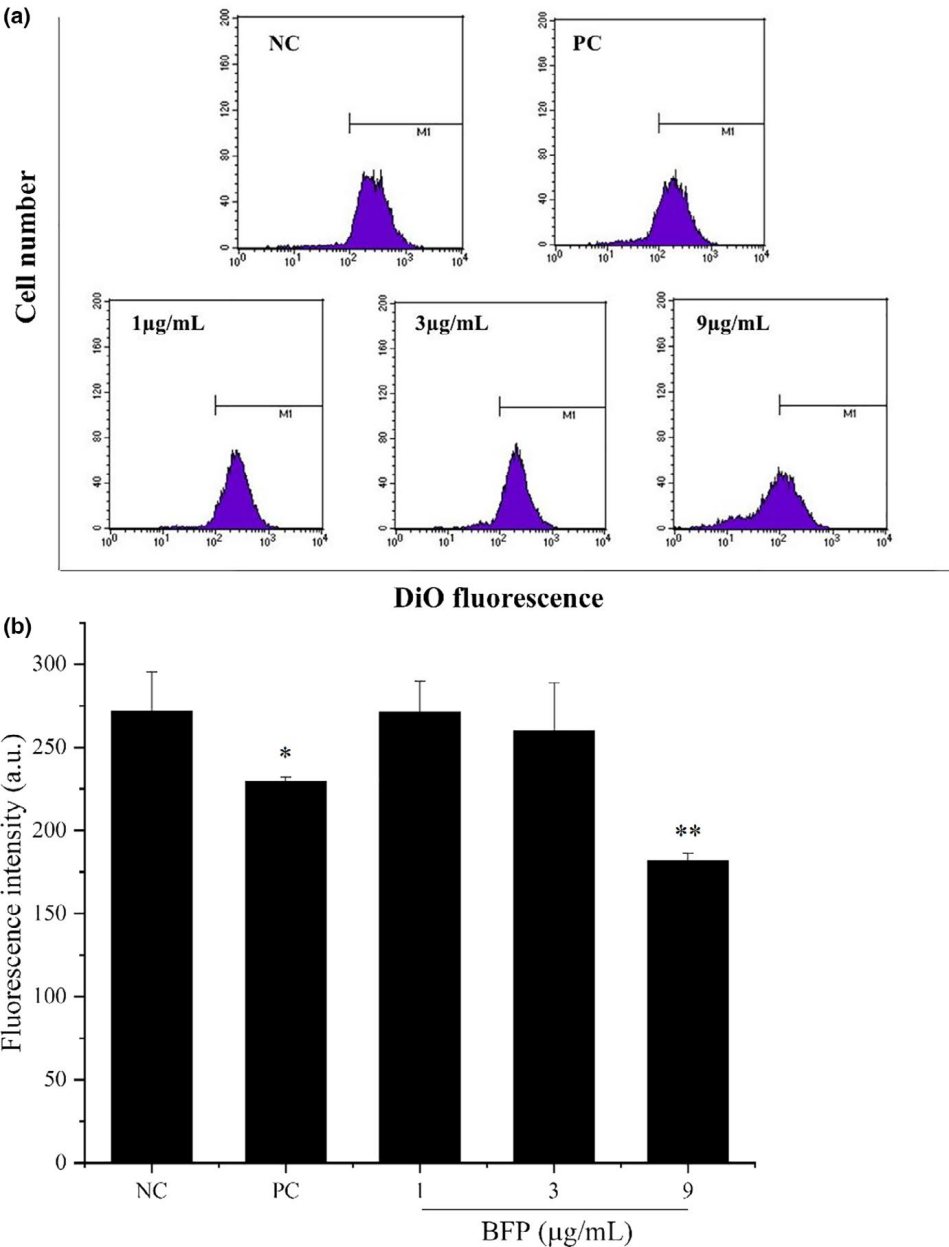
Effect of BFP treatment on the mitochondrial membrane potential (MMP) of A2780 cells. Cells were subjected to treatment with 0.01 µg/mL taxol (positive control, PC) and BFP (1, 3, and 9 µg/mL), and a negative control group (NC) was established. (a) MMP was evaluated by flow cytometry after staining the cells with DiO. (b) Quantification of MMP. *p < .05 and **p < .01 versus. NC group (mean ± SD, *n* = 3)

One of the hallmark changes occurring during early apoptosis is a decrease in MMP. Figure 4 showed that treatment with the highest BFP dose (9 μg/mL) caused a significant reduction in the MMP compared with that in the NC group (*p* < .01).

### 3.5 Effect of BFP treatment on ROS production in A2780 cells

**FIGURE 5:**
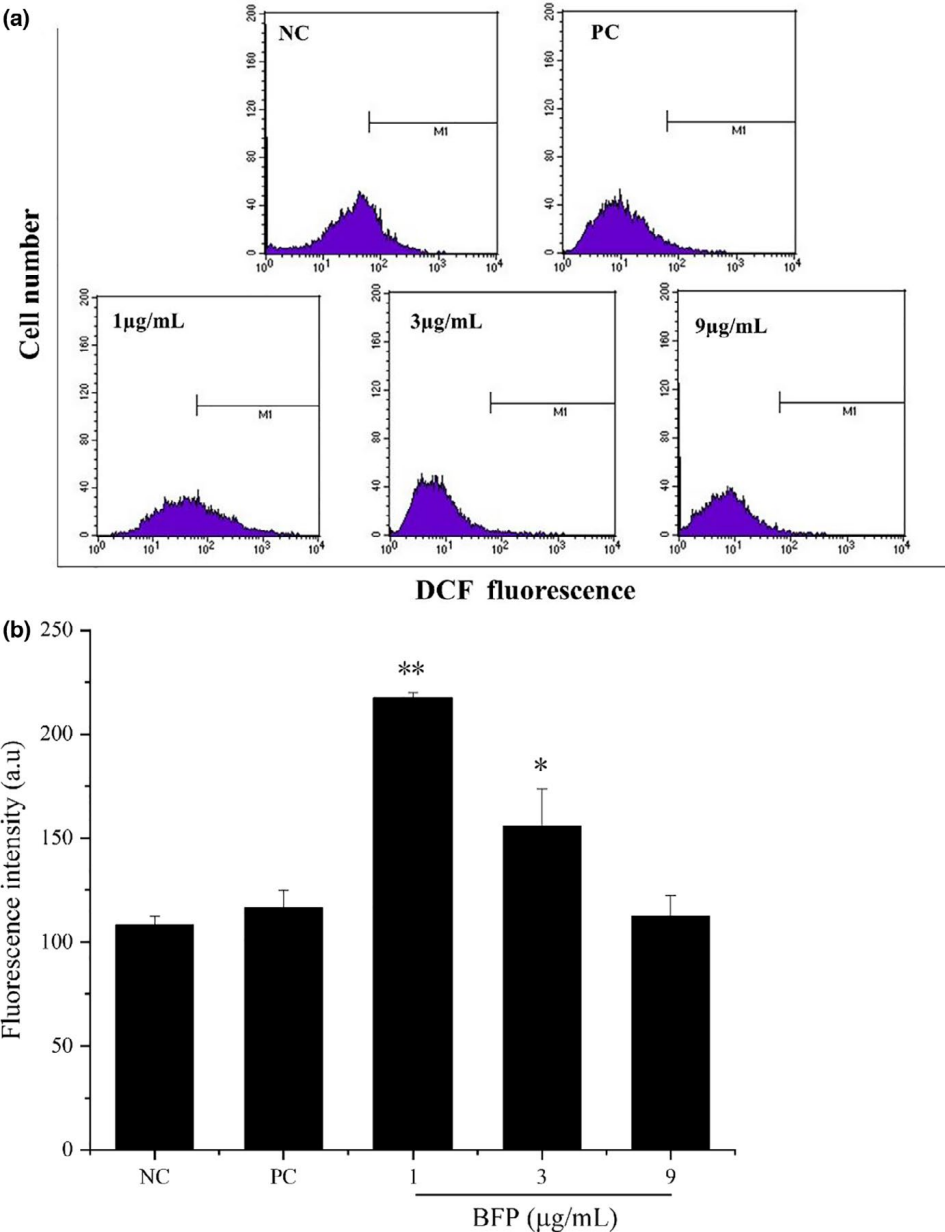
Effect of BFP treatment on reactive oxygen species (ROS) generation in A2780 cells. Cells were subjected to treatment with 0.01 µg/mL taxol (positive control, PC) and BFP (1, 3, and 9 µg/mL), and a negative control group (NC) was established. (a) Reactive oxygen species (ROS) production was measured using flow cytometry after staining the cells with 2′,3′‐dichlorodihydrofluorescein diacetate (DCFH‐DA). (b) Quantification of ROS production. *p < .05 and **p < .01 versus. NC group (mean ± SD, *n* = 3)

Excessive ROS levels eventually affect MMP and inflict damage upon the mitochondria, leading to cellular autophagy and/or apoptosis (Chang et al., 2017). Incubation of A2780 cells with BFP (1 μg/mL) for 24 h significantly increased intracellular ROS production (*p* < .01), as represented by the increased DCF fluorescence. However, ROS production declined with increasing BFP concentrations (Figure 5). Additionally, there was no evident change in ROS production after subjection to taxol (PC) treatment.

### 3.6 Effects of BFP treatment on apoptosis‐related mRNA and protein expression

**FIGURE 6:**
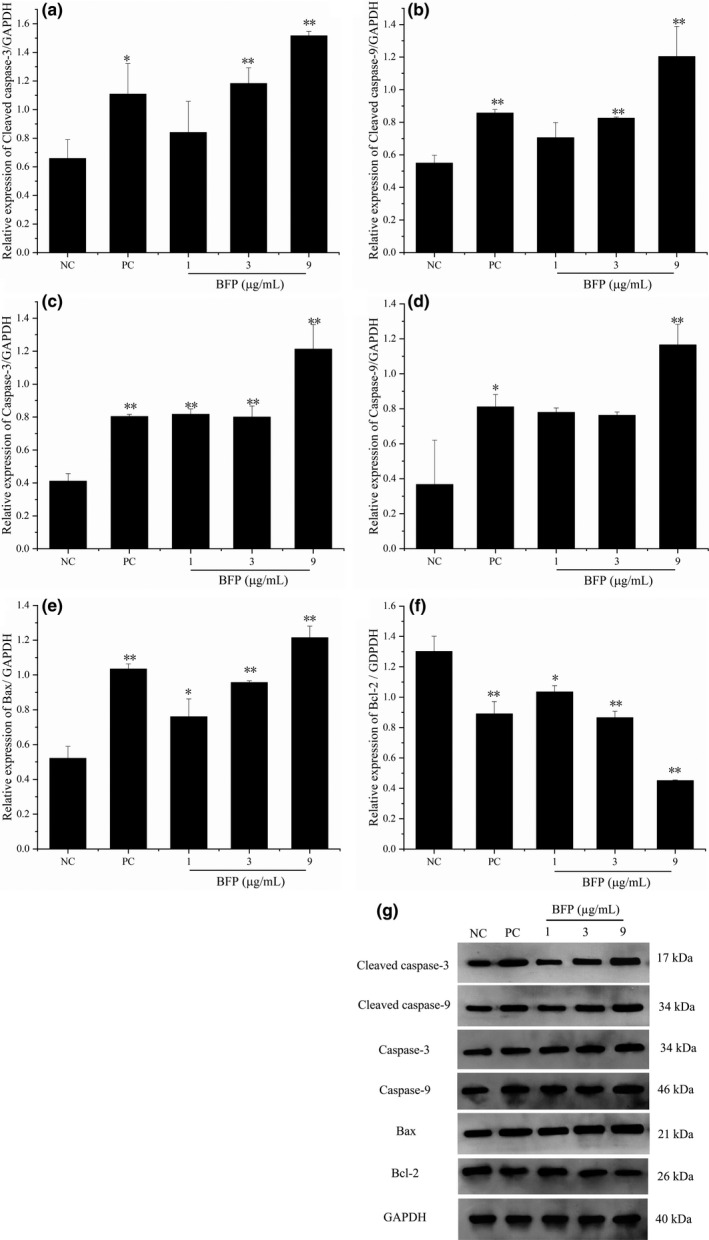
Effect of BFP treatment on the expression of cleaved caspase‐3 (C‐caspase‐3), cleaved caspase‐9 (C‐caspase‐9), caspase‐3, caspase‐9, BAX, and BCL2 in A2780 cells. A2780 cells were subjected to treatment with 0.01 µg/mL taxol (positive control, PC) and BFP (1, 3, and 9 µg/mL), and a negative control (NC) group was established. (a–f) Quantitative analysis of C‐caspase‐3, C‐caspase‐9, caspase‐3, caspase‐9, BAX, and BCL2 proteins. (g) Expression of C‐caspase‐3, C‐caspase‐9, caspase‐3, caspase‐9, BAX, and BCL2 proteins was confirmed using Western blotting, and glyceraldehyde‐3‐phosphate dehydrogenase (GAPDH) was used as a loading control. *p < .05 and **p < .01 versus. NC group (mean ± SD, *n* = 3)

To investigate the role of the BCL2 family in BFP‐induced apoptosis, we first analyzed the changes in the levels of anti‐apoptotic BCL2 and pro‐apoptotic BAX. qRT–PCR (Table 2) and Western blot (Figure 6) analyses revealed that treatment of A2780 cells with BFP increased pro‐apoptotic BAX expression in the cells in a dose‐dependent manner, whereas anti‐apoptotic BCL2 expression was significantly downregulated after similar treatment. At 9 µg/mL BFP, the relative mRNA expression levels of *BAX* and *BCL2* were 5.22‐ and 0.18‐fold than those in the NC group, whereas the relative protein expression levels were 2.33‐ and 0.34‐fold than those in the NC group, respectively. Thus, the ratio of *BAX* to *BCL2* increased substantially after BFP treatment, thus promoting apoptosis.

**TABLE 2:** mRNA expression of *CASP3, CASP9, BAX*, and *BCL2* in A2780 cells subjected to treatment with polysaccharides isolated from *Bangia fuscopurpurea* (BFP)

Group	*CASP3*	*CASP9*	*BAX*	*BCL2*
1	1.0046 ± 0.0434	1.1993 ± 0.2102	1.2847 ± 0.3138	1.2398 ± 0.1985
2	3.8242 ± 1.0739*	3.1890 ± 0.5718**	3.0558 ± 0.6092**	0.2982 ± 0.0149**
3	1.7624 ± 0.5899	2.1393 ± 0.6169	2.3755 ± 0.0775**	0.7035 ± 0.1182*
4	3.7907 ± 1.3463*	2.9620 ± 0.4067**	3.4231 ± 0.4241**	0.3866 ± 0.0569**
5	5.2498 ± 0.7110**	4.1173 ± 0.9621**	5.4361 ± 1.4126**	0.2299 ± 0.0520**

Note

1. Negative control (NC) group; 2. A2780 cells subjected to treatment with 0.01 µg/mL taxol (positive control group, PC); 3. A2780 cells subjected to treatment with 1 µg/mL BFP; 4. A2780 cells subjected to treatment with 3 µg/mL BFP; 5. A2780 cells subjected to treatment with 9 µg/mL BFP. *p < .05 and **p < .01 versus. NC group.

Considering that the caspase family plays a central role in the apoptotic process, we examined the effect of BFP treatment on caspase activation. BFP treatment remarkably increased the transcription and protein expression of cleaved caspase‐3, cleaved caspase‐9, caspase‐3, and caspase‐9 in A2780 cells (Table 2, Figure 6). At 9 µg/mL BFP, the relative mRNA expression levels of caspase‐3 and caspase‐9 were 3.43‐ and 4.23‐fold than those in the NC, whereas the protein expression levels of cleaved caspase‐3, cleaved caspase‐9, caspase‐3, and caspase‐9 were 2.30‐, 2.19‐, 2.95‐, and 3.17‐fold than those in the NC group, respectively. Our results suggested that BFP induced apoptosis by changing the BAX/BCL2 ratio, thereby increasing mitochondrial membrane permeability to trigger an entire cascade of apoptotic reactions.

### 3.7 Effect of BFP treatment on autophagy in A2780 cells

**FIGURE 7:**
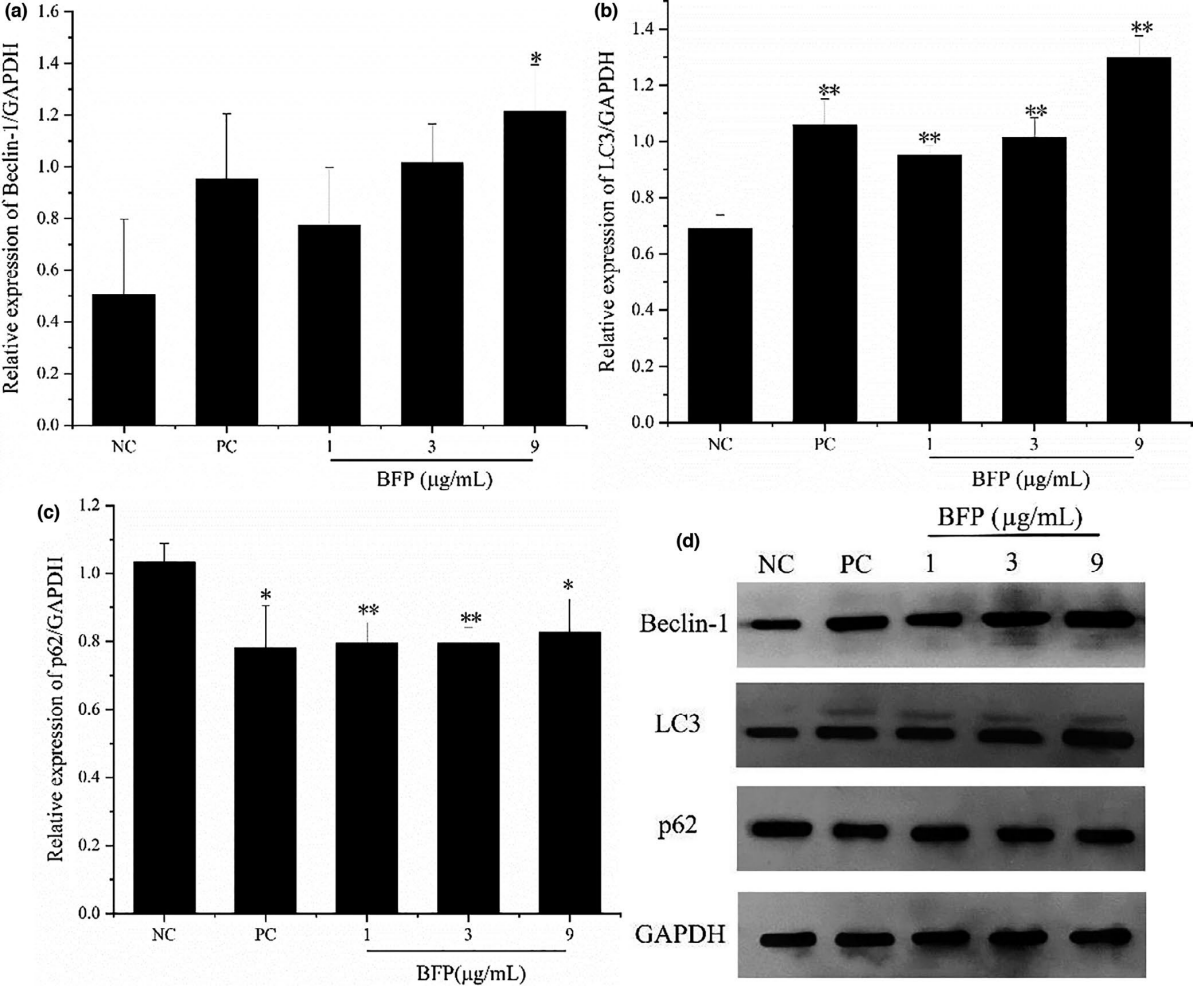
Effect of BFP treatment on the protein expression of LC3, Beclin‐1, and P62 in A2780 cells. The cells were subjected to treatment with 0.01 µg/mL taxol (positive control, PC) and BFP (1, 3, and 9 µg/mL), and a negative control (NC) group was established. (a–c) Quantitative analysis of LC3, Beclin‐1, and P62 proteins. (d) Expression of LC3, Beclin‐1, and P62 proteins was confirmed using Western blotting, and GAPDH was used as a loading control. *p < .05 and **p < .01 versus. NC group (mean ± SD, *n* = 3)

To determine whether autophagy was involved in BFP‐induced A2780 cell death, autophagy‐related mRNA and protein expression were examined after the cells were subjected to treatment with various concentrations of BFP. The dose‐dependent effects of 24 h BFP treatment on LC3 distribution were detected using qRT–PCR and Western blotting analyses. LC3 levels increased in a dose‐dependent manner after BFP treatment (Table 3, Figure 7), indicating a cumulative increase in autophagosome formation as BFP stimuli progressed. This was confirmed by the significant increase in the expression of Beclin‐1, an essential protein in autophagosome formation, in BFP‐treated cells (Table 3, Figure 7). However, the appearance of more autophagosomes did not necessarily equate with more autophagy. Autophagosome accumulation may be caused by a block in trafficking to lysosomes, hence the reduction in degradative activity. Therefore, we next examined P62 expression, which serves as an index of autophagic degradation, and found a significant decrease in its expression upon BFP treatment compared with the NC group (*p* < .01, *p* < .05), suggesting that the delivery of cargo to lysosomes was not blocked and that autophagic degradation was enhanced after BFP treatment.

**TABLE 3:** mRNA expression of *BECN1, LC3*, and *P62* in A2780 cells subjected to treatment with polysaccharides isolated from *Bangia fuscopurpurea* (BFP)

Group	*BECN1*	*LC3*	*P62*
1	1.0499 ± 0.0451	1.2167 ± 0.1262	1.2623 ± 0.1518
2	12.4608 ± 1.5463**	3.2006 ± 0.6085**	0.6623 ± 0.0991**
3	4.7833 ± 1.2348**	1.9265 ± 0.3857*	0.8466 ± 0.0223*
4	8.0037 ± 1.1026**	3.4142 ± 0.5159**	0.7390 ± 0.0568**
5	14.3784 ± 1.6741**	5.6413 ± 1.2327**	0.6715 ± 0.0485**

Note

1. Negative control (NC) group; 2. A2780 cells subjected to treatment with 0.01 µg/mL taxol (positive control group, PC); 3. A2780 cells subjected to treatment with 1 µg/mL BFP; 4. A2780 cells subjected to treatment with 3 µg/mL BFP; 5. A2780 cells subjected to treatment with 9 µg/mL BFP. *p < .05 and **p < .01 versus. NC group.

**FIGURE 8:**
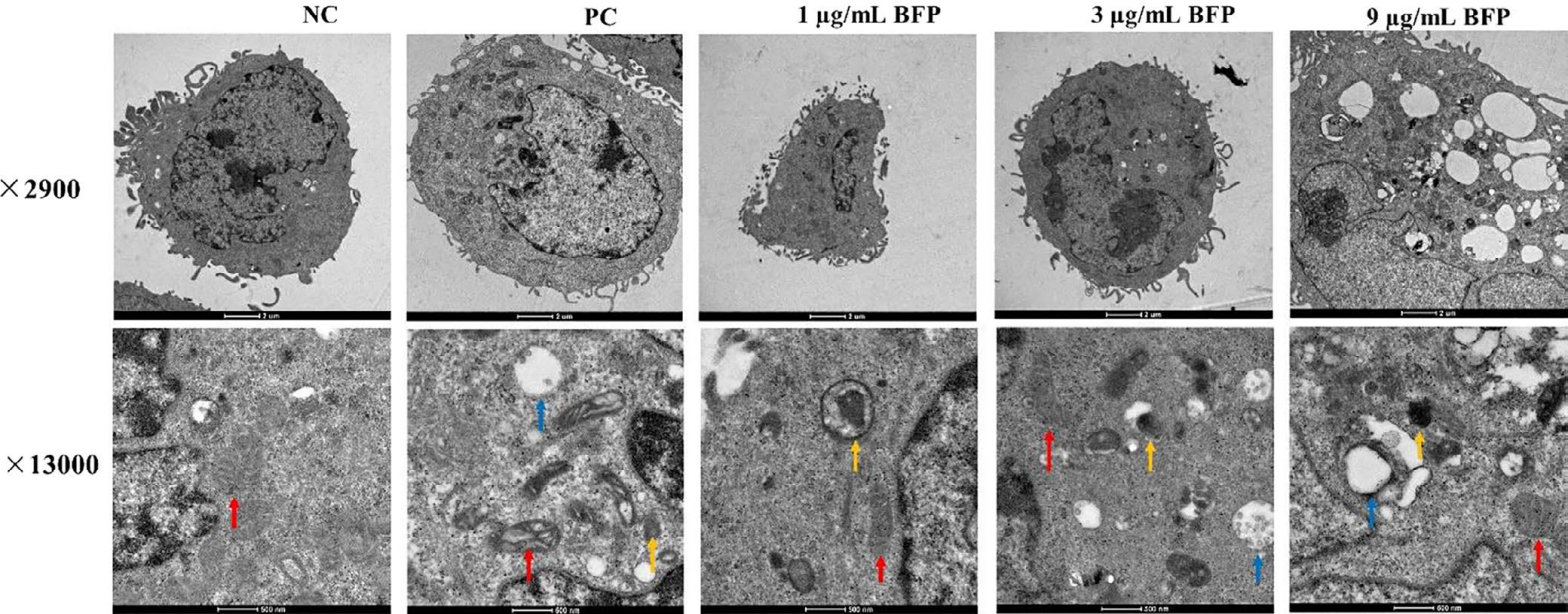
Transmission electron microscopy (TEM) analysis of A2780 cells. Cells were subjected to treatment with 0.01 µg/mL taxol (positive control, PC) and BFP (1, 3, and 9 µg/mL), and a negative control (NC) group was established. Mitochondrion, red arrow; lysosome, blue arrow; autophagosome, yellow arrow

Furthermore, TEM was conducted to investigate BFP‐mediated stimulation of autophagosome formation and its fusion with lysosomes in A2780 cells via morphology analysis (Figure 8). Autophagic vacuoles with a unique double membrane could be observed in A2780 cells subjected to treatment with BFP for 24 h. Although there were few autophagic vacuoles in the NC group, the cells and membranes remained intact; mitochondrial morphology was normal; inner ridges were clear; autophagosomes were infrequent; and overall structure was complete. In the 1 μg/mL BFP‐treated group, the mitochondrial morphology changed slightly, inner ridges were observed, and the number of autophagosomes increased. In the 3 μg/mL BFP‐treated group, the ridges in the mitochondria disappeared; mitochondria were lysed; lysosomes were degraded; and a substantial number of autophagosomes were observed. In the 9 μg/mL BFP‐treated group, the mitochondria were degraded and autophagosomes were abundant.

## 4 DISCUSSION

Ovarian cancer is the most fatal gynecologic malignancy due to its characteristic late diagnosis, and patients in the metastatic stage have limited effective options available for therapies (Chen et al., 2014). Therefore, identification of new agents with improved, non‐toxic antitumor effects is necessary. The anti‐cancer potential of any compound primarily depends on its ability to reduce the viability and proliferation of cancer cells. We have previously shown that BFP treatment reduces the viability of A2780 cells in a dose‐dependent manner, with a 50% reduction (IC_50_) in viability at 23.69 μg/mL BFP (Wu et al., 2021). In the present study, we explored the anti‐cancer effects of BFP on ovarian cancer cells in vitro and demonstrated their underlying mechanisms. BFP exerted an antitumor effect of ovarian cancer, as evidenced by inhibited cell migration and invasion, and promoted apoptotic and autophagic cell death via the mitochondria‐dependent pathway. Therefore, BFP is a potential therapeutic candidate for ovarian cancer.

The underlying anti‐cancer mechanisms of algal polysaccharides include the induction of apoptosis and autophagy, cell cycle arrest, suppression of migration and angiogenesis, modulation of transduction signaling pathways, and activation of immune responses and the antioxidant system (Li et al., 2017; Sajadimajd et al., 2019). Taxol, one of the broadest‐spectrum anti‐cancer agents available, is currently used to treat patients with ovarian, breast, and non‐small cell lung cancers. Taxol inhibits the migration and promotes the death of cancer cells by inducing anti‐apoptotic molecule inactivation, by inhibiting oncogene expression, by activating immune cells, and by regulating microtubule stability kinetics (Feng & Mumper, 2013; Schiff et al., 1979; Sun et al., 2018). Therefore, we selected cells subjected to treatment with 0.01 μg/mL taxol as our PC group. We found that BFP and taxol treatment effectively inhibited the migration and invasion of A2780 cells. Cells subjected to treatment with taxol underwent arrest in the G2/M phase, and this finding was consistent with that reported in previous studies (Kumaran et al., 2009; Wang et al., 2000). Contrastingly, there was no evident dose relationship between BFP and the cell cycle, possibly due to the different degrees of apoptosis. Hence, BFP treatment did not seem to prominently affect the cell cycle.

Apoptosis is the key target of most chemotherapeutic agents (Ling et al., 2011). We quantified the number of apoptotic cells upon BFP treatment to evaluate the loss of plasma membrane integrity (Wlodkowic et al., 2009). Our results corroborate the apoptosis event. In a previous study, most polysaccharides followed the mitochondrial pathway, one of the two pathways of apoptosis (Jose et al., 2018). A decrease in MMP resulting from mitochondrial membrane depolarization indicates mitochondrial damage, an initial and irreversible step in apoptosis (Chen et al., 2019). To confirm the involvement of this pathway in the anti‐cancer activity of BFP, the MMP of the A2780 cells was determined; a significant dose‐dependent decrease in the MMP was observed in the cells. Several polysaccharides have been shown to induce a decrease in MMP, leading to the translocation of cytochrome C from the mitochondria to the cytoplasm, thereby activating a cascade of caspases, a group of cysteine proteases, and ultimately triggering apoptosis. After treatment with 0.25, 0.50, and 1.00 mg/mL LGPS‐1, a novel neutral polysaccharide isolated from *Lentinus giganteus*, MMP collapse was induced, and cytochrome C expression was increased in HepG2 cells (Tian et al., 2016). Similar observations were reported for HeLa cells subjected to treatment with 1.2 mg/mL of ESP‐CP that are sulfated polysaccharides derived from *Padina tetrastromatica* (Jose et al., 2018). Consistent with these reports, we observed that BFP‐induced apoptosis in A2780 cells was accompanied by MMP depolarization.

In addition to disrupting the MMP, few polysaccharides stimulated ROS generation, which might have played a role in enhancing their anti‐cancer activity (Khan et al., 2019). Apoptosis can be induced by ROS accumulation in live cells, and this has been implicated in the activation of various transcriptional factors and eventual apoptosis (Hseu et al., 2019; Pan et al., 2015). Treating A2780 cells with NAC (1 μM), an ROS inhibitor, and BFP (1, 3, and 9 μg/mL) resulted in significant different rates of apoptosis. This finding suggests that apoptosis is ROS‐dependent. Here, we monitored the ROS levels in A2780 cells after BFP treatment to determine whether apoptosis was induced by ROS. However, ROS production declined with increasing BFP concentrations, and there was no substantial change in ROS production after taxol treatment, in contrast to a previous report which showed the occurrence of mitochondrial permeability transition and ROS formation after administration of taxol (Varbiro et al., 2001). Thus, ROS may not have been detected because of the higher drug concentrations used and consequent high rate of apoptosis.

To further explore the detailed mitochondrial apoptosis pathway, apoptosis‐related mRNA and protein expression were evaluated. BAX and BCL2 are two important BCL2 family proteins that regulate apoptosis (Jose et al., 2018), and the regulation also involves activation of members of the caspase protein family (Wang et al., 2019). Notably, the BCL2 family comprises both anti‐apoptotic and pro‐apoptotic proteins, which are essential for the regulation of mitochondrial apoptosis; therefore, they should be considered as major mediators of the mitochondrial apoptotic pathway (Zhang et al., 2015). In this pathway, cytochrome C is released, and caspase‐9 is activated, leading to activation of caspase‐3 and apoptosis (Yu et al., 2020). Our results indicated that BFP treatment promoted cytochrome C release by upregulating BAX expression, by downregulating BCL2 expression, and by activating caspase‐3 and caspase‐9 proteins by cleaving them to pro‐caspase, suggesting that BFP treatment activated the mitochondrial apoptosis pathway in A2780 cells.

Autophagy, another self‐destructive process that involves the action of lysosomes to degrade damaged organelles and macromolecular substances plays dual roles in cancer progression (Zhao et al., 2017). It plays a protective role under stressful conditions such as starvation, radiation exposure, or chemical insults. Additionally, autophagy is activated as a protective mechanism in chemotherapy resistance. Furthermore, excessive autophagy stimulates the destruction of malignant cells. In fact, autophagy is an alternative mechanism that promotes death of cancer cells with natural defects in apoptosis (Peng et al., 2018). Therefore, regulation of autophagy to promote cell death is a potential therapeutic approach for cancer treatment. Overexpression of Beclin‐1, the presence of LC3 in autophagosomes, and conversion of LC3‐I to LC3‐II are known indicators of autophagy (Liu et al., 2017). P62 is incorporated into the completed autophagosome, which is then degraded by autolysosomes and can serve as an index of autophagic degradation (Li, Chen, et al., 2018). Our results showed that the expression of Beclin‐1 and LC3 was enhanced, whereas that of P62 was reduced upon BFP treatment. Moreover, an increased number of autophagic vacuoles with a double membrane structure was observed in BFP‐treated cells. Overall, these results demonstrate excessive activation of autophagy in A2780 cells after BFP treatment.

The extent of the relationship between autophagy and apoptosis is complex and not completely understood. Both pathways have common upstream triggers (e.g., ROS, Ca^2+^, and DNA damage) and share common components. ROS can induce mitochondria‐mediated apoptosis and stimulate the proteolytic activity of ATG4, a cysteine protease necessary for autophagy, thereby stimulating autophagy (Maiuri et al., 2007; Mukhopadhyay et al., 2014). Anti‐apoptotic BCL2 establishes interactions with Beclin‐1 and BAX/BAK to regulate autophagy and apoptosis (Patra et al., 2020). The mutual interaction between the proteins regulating apoptosis and autophagy may mediate the switchover.

## 5 CONCLUSION

We determined the possible mechanism underlying the potential anti‐cancer properties of BFP in vitro using the human ovarian cancer cell line A2780. This mechanism seems to involve the suppression of cancer cell migration and invasion and promotion of apoptotic and autophagic cell death via the mitochondria‐dependent pathway. It is generally accepted that autophagy can inhibit apoptosis and confer protection to cells. Importantly, autophagy always occurs before the initiation of apoptosis. However, autophagy can also lead to apoptosis and promote cell death. In our study, we did not explore the relationship between apoptosis and autophagy induced by BFP, and thus their mechanisms remain unclear. Additionally, the cell signaling pathways of BFP‐induced A2780 cell apoptosis and autophagy are unknown and should be further investigated. Overall, our results suggest BFP may be a potential therapeutic candidate for ovarian cancer.

## CONFLICT OF INTEREST

The authors declare no conflict of interest.
